# Development and Validation of a Novel COVID-19 nsp8 One-Tube RT-LAMP-CRISPR Assay for SARS-CoV-2 Diagnosis

**DOI:** 10.1128/spectrum.01962-22

**Published:** 2022-11-29

**Authors:** Cyril Chik-Yan Yip, Siddharth Sridhar, Wan-Mui Chan, Jonathan Daniel Ip, Allen Wing-Ho Chu, Kit-Hang Leung, Vincent Chi-Chung Cheng, Kwok-Yung Yuen, Kelvin Kai-Wang To

**Affiliations:** a Department of Microbiology, Queen Mary Hospital, Pokfulam, Hong Kong Special Administrative Region, China; b State Key Laboratory for Emerging Infectious Diseases, Carol Yu Centre for Infection, Department of Microbiology, School of Clinical Medicine, Li Ka Shing Faculty of Medicine, The University of Hong Kong, Pokfulam, Hong Kong Special Administrative Region, China; c Department of Clinical Microbiology and Infection, The University of Hong Kong -Shenzhen Hospital, Shenzhen, Guangdong Province, China; d Centre for Virology, Vaccinology and Therapeutics, Hong Kong Science and Technology Park, Sha Tin, Hong Kong Special Administrative Region, China; University of Prince Edward Island

**Keywords:** SARS-CoV-2, nsp8, RT-LAMP, CRISPR, diagnostic assay

## Abstract

Accurate and simple diagnostic tests for coronavirus disease 2019 (COVID-19) are essential components of the pandemic response. In this study, we evaluated a one-tube reverse transcription–loop-mediated isothermal amplification (RT-LAMP) assay coupled with clustered regularly interspaced short palindromic repeat (CRISPR)-associated protein-mediated endpoint detection of severe acute respiratory syndrome coronavirus 2 (SARS-CoV-2) RNA in clinical samples. RT-LAMP-CRISPR is fast and affordable, does not require bulky thermocyclers, and minimizes carryover contamination risk. Results can be read either visually or with a fluorometer. RT-LAMP-CRISPR assays using primers targeting a highly expressed nsp8 gene and previously described nucleocapsid (N) gene primers were designed. The analytical characteristics and diagnostic performance of RT-LAMP-CRISPR assays were compared to those of a commercial real-time RT-PCR E gene assay. The limits of detection (LODs) of the nsp8 and N RT-LAMP-CRISPR assays were 750 and 2,000 copies/mL, which were higher than that of the commercial real-time RT-PCR assay (31.3 copies/mL). Despite the higher LOD, RT-LAMP-CRISPR assays showed diagnostic sensitivity and specificity of 98.6% and 100%, respectively, equivalent to those of the real-time RT-PCR assay (*P* = 0.5). The median fluorescence reading from the nsp8 assay (378.3 raw fluorescence unit [RFU] [range, 215.6 to 592.6]) was significantly higher than that of the N gene assay (342.0 RFU [range, 143.0 to 576.6]) (*P* < 0.0001). In conclusion, we demonstrate that RT-LAMP-CRISPR assays using primers rationally designed from highly expressed gene targets are highly sensitive, specific, and easy to perform. Such assays are a valuable asset in resource-limited settings.

**IMPORTANCE** Accurate tests for the diagnosis of SARS-CoV-2, the virus causing coronavirus disease 2019 (COVID-19), are important for timely treatment and infection control decisions. Conventional tests such as real-time reverse transcription-PCR (RT-PCR) require specialized equipment and are expensive. On the other hand, rapid antigen tests suffer from a lack of sensitivity. In this study, we describe a novel assay format for the diagnosis of COVID-19 that is based on principles of loop-mediated isothermal amplification (LAMP) and clustered regularly interspaced short palindromic repeat (CRISPR)-Cas chemistry. A major advantage of this assay format is that it does not require expensive equipment to perform, and results can be read visually. This method proved to be fast, easy to perform, and inexpensive. The test compared well against an RT-PCR assay in terms of the ability to detect SARS-CoV-2 RNA in clinical samples. No false-positive test results were observed. The new assay format is ideal for SARS-CoV-2 diagnosis in resource-limited settings.

## INTRODUCTION

The coronavirus disease 2019 (COVID-19) pandemic, caused by severe acute respiratory syndrome coronavirus 2 (SARS-CoV-2), has entered its third year (1–3). With more than 630 million confirmed cases worldwide, it is already one of the largest pandemics in recorded history (https://www.who.int/emergencies/diseases/novel-coronavirus-2019) (4). Prompt COVID-19 diagnosis is a central pillar of pandemic control because this not only guides infection control in community and health care settings but also informs early treatment decisions for at-risk individuals (5–8). Globally, COVID-19 diagnosis relies mostly on antigen tests (usually in a lateral flow format) or nucleic acid amplification tests (NAATs) such as reverse transcription-PCR (RT-PCR) (9). Antigen tests are relatively inexpensive and can be used as point-of-care tests but suffer from a lack of sensitivity for early COVID-19 diagnosis (10, 11). In contrast, RT-PCR is highly sensitive but requires centralized testing in a laboratory equipped with trained staff and specialized equipment such as thermocyclers (12–14). Therefore, RT-PCR cannot be conveniently deployed at scale in resource-limited settings.

Isothermal NAAT methods such as loop-mediated isothermal amplification (LAMP) obviate a thermocycler by amplifying the target nucleic acid at a constant temperature (15). LAMP product detection can be performed using CRISPR-Cas (clustered regularly interspaced short palindromic repeat–CRISPR-associated protein) chemistry (16). CRISPR-Cas systems are critical components of bacterial immunity that selectively cleave nucleic acids of invaders like bacteriophages (17). CRISPR effector proteins like Cas12a selectively cleave DNA when activated by guide RNA (gRNA) (18). This feature can be exploited for the specific detection of the end products of LAMP.

In this study, we describe the design and evaluation of a novel one-tube reverse transcription-LAMP (RT-LAMP)-CRISPR assay for COVID-19 diagnosis. In our previous study, two highly expressed gene regions, PB2 and NS of influenza A viruses, were successfully identified by nanopore sequencing and used as the targets for the development of a highly sensitive molecular assay for influenza A virus detection (19). We used the same strategy to identify highly expressed gene regions of SARS-CoV-2 for the development of the RT-LAMP-CRISPR assay (20). Several studies demonstrated SARS-CoV-2 detection by RT-LAMP and CRISPR-Cas12a using lateral flow strips (21–23), which involved opening tubes for combining RT-LAMP products with the CRISPR reagent followed by the strip readout. This would increase the chance of amplicon contamination. To reduce the risk of contamination, we developed the assay in a one-tube format. Another aim of our study is to reduce the cost of each reaction without sacrificing the sensitivity of the assay, and therefore we attempted to reduce the amount of RT-LAMP reagents compared to those used in other studies (22, 24, 25). This assay is rapid, sensitive, specific, affordable, and user-friendly. In addition, it does not require thermocyclers, enabling it to be deployed at scale even in resource-limited settings.

## RESULTS

### Design of a novel COVID-19 RT-LAMP-CRISPR assay targeting the nsp8 gene of the SARS-CoV-2 genome in a one-tube format.

Our previous study showed that in addition to the nsp1 and nucleocapsid (N) genes, the nsp8 gene of SARS-CoV-2 was also highly expressed in clinical specimens, as revealed by nanopore whole-genome sequencing with the nanopore protocol for PCR tiling of COVID-19 (version PTC_9096_v109_revH_06Feb2020) (see Fig. S1 in the supplemental material) (Global Initiative on Sharing All Influenza Data [GISAID] accession number EPI_ISL_450409) (20). Therefore, we designed primers targeting the nsp8 gene region as a novel target for the RT-LAMP-CRISPR assay (Table 1) using the New England BioLabs (NEB) LAMP primer design tool (https://lamp.neb.com/#!/). The multiple-sequence alignment showed that the target sites of our nsp8 primers and gRNA were well conserved among different variants (ancestral strain, Alpha, Beta, Gamma, Delta, Lambda, Mu, and Omicron [BA.1, BA.2, BA.3, BA.4, and BA.5]) (see Fig. S2 in the supplemental material). As a comparator, we chose the N gene primers designed previously by Broughton et al. (21).

**TABLE 1:** Primers used for RT-LAMP reactions in this study

Target (reference[s]) and primer	Sequence (5′–3′)
nsp8 gene of SARS-CoV-2 (this study)	
SARS-CoV-2_nsp8_F3	TGGATAATGATGCACTCAACAACA
SARS-CoV-2_nsp8_B3	TGTCCATACTAATTTCACTAAGTTGA
SARS-CoV-2_nsp8_FIP	ACCATTAGTTTGGCTGCTGTAAGAGATGGTTGTGTTCCCT
SARS-CoV-2_nsp8_BIP	ACGTGTGATGGTACAACATTTACTCTGCATCTACAACCTGTTGGATT
SARS-CoV-2_nsp8_LB	TGCATCAGCATTGTGGGA
N gene of SARS-CoV-2 (21, 24)	
SARS-CoV-2_N_F3	AACACAAGCTTTCGGCAG
SARS-CoV-2_N_B3	GAAATTTGGATCTTTGTCATCC
SARS-CoV-2_N_FIP	TGCGGCCAATGTTTGTAATCAGCCAAGGAAATTTTGGGGAC
SARS-CoV-2_N_BIP	CGCATTGGCATGGAAGTCACTTTGATGGCACCTGTGTAG
SARS-CoV-2_N_LF	TTCCTTGTCTGATTAGTTC
SARS-CoV-2_N_LB	ACCTTCGGGAACGTGGTT
Human RNase P gene (21)	
RNaseP-POP7_F3	TTGATGAGCTGGAGCCA
RNaseP-POP7_B3	CACCCTCAATGCAGAGTC
RNaseP-POP7_FIP	GTGTGACCCTGAAGACTCGGTTTTAGCCACTGACTCGGATC
RNaseP-POP7_BIP	CCTCCGTGATATGGCTCTTCGTTTTTTTCTTACATGGCTCTGGTC
RNaseP-POP7_LF	ATGTGGATGGCTGAGTTGTT
RNaseP-POP7_LB	CATGCTGAGTACTGGACCTC

In the present study, the one-tube-format RT-LAMP-CRISPR assay was achieved by mixing a sample nucleic acid extract with an RT-LAMP reagent mixture at the bottom of a PCR tube and adding a Cas12a reagent mixture inside the cap of the tube (24). After the RT-LAMP reaction, the Cas12a reagent mixture was flicked to the bottom of the tube and mixed with the RT-LAMP amplicon without opening the tube for CRISPR. Fluorescence could be visualized under UV excitation or measured using a fluorometer.

### Analytical sensitivity and specificity of the novel nsp8 gene and the comparative nucleocapsid gene RT-LAMP-CRISPR assays for SARS-CoV-2 detection.

To compare the analytical sensitivities of the nsp8 and N gene RT-LAMP-CRISPR assays, the limit of detection (LOD) was evaluated by using 2-fold serial dilutions of total nucleic acid (TNA) extracted from SARS-CoV-2 Q control 01, which is a positive control with a target SARS-CoV-2 concentration of 10,000 copies/mL. The LODs of the nsp8 and N assays were 750 and 2,000 copies/mL (equivalent to 15 and 40 copies/reaction), respectively (Table 2). The LOD of the commercial LightMix E gene real-time RT-PCR assay was 31.3 copies/mL (equivalent to 1.3 copies/reaction) (Table 2). Both the nsp8 and N gene RT-LAMP-CRISPR assays showed no cross-reaction with SARS-CoV-1 and other common respiratory viruses.

**TABLE 2:** Test results for determining the limits of detection of the in-house-developed nsp8 and N gene RT-LAMP-CRISPR assays and the commercial LightMix E gene RT-PCR assay with TNA extracted from Qnostics SARS-CoV-2 Q control 01^*a*^

Concn (copies/mL)	Result					
	Intrarun			Interrun		
	Test 1	Test 2	Test 3	Test 1	Test 2	Test 3
nsp8 gene RT-LAMP-CRISPR assay						
1,000	+	+	+	+	+	+
750	+	+	+	+	+	+
500	+	+	+	−	−	−
250	+	−	−	−	−	−
N gene RT-LAMP-CRISPR assay						
4,000	+	+	+	+	+	+
2,000	+	+	+	+	+	+
1,000	+	+	+	+	+	−
500	−	−	−	+	+	+
LightMix E gene RT-PCR assay						
125	+	+	+	+	+	+
62.5	+	+	+	+	+	+
31.3	+	+	+	+	+	+
15.6	+	−	−	+	+	−

a +, detected; −, not detected.

### Evaluation of the diagnostic performance of the nsp8 and N gene RT-LAMP-CRISPR assays for SARS-CoV-2 detection using sample extracts.

To assess the diagnostic performance of the nsp8 and N gene RT-LAMP-CRISPR assays using clinical specimens, we selected a total of 319 respiratory specimens, including 149 nasopharyngeal and 170 saliva specimens, which were previously tested with the commercial LightMix E gene RT-PCR assay. Among these specimens, 46.4% (148/319) tested positive for SARS-CoV-2 by LightMix E gene RT-PCR (median crossing point [Cp] value, 23.1 [range, 14.2 to 36.6]), while 45.8% (146/319) tested positive for SARS-CoV-2 by both the nsp8 and N gene RT-LAMP-CRISPR assays (Table 3). There was no significant difference in the rates of detection between the in-house nsp8/N gene RT-LAMP-CRISPR assay and the commercial E gene RT-PCR assay for saliva or all specimens (*P* = 0.5) and nasopharyngeal specimens (*P* = 1) (Table 3). To monitor the presence of cellular material in the clinical specimens, primers targeting the human RNase P gene (Table 1) were used as internal controls (21). Fluorescence was detected for all of the available sample extracts by the RNase P RT-LAMP-CRISPR assay, proving the validity of the tested specimens (see Fig. S3 in the supplemental material). Using the LightMix E gene RT-PCR assay as the reference method, the diagnostic sensitivity and specificity of both the nsp8 and N gene RT-LAMP-CRISPR assays were 98.6% and 100%, respectively. The sensitivity of both the nsp8 and N gene RT-LAMP-CRISPR assays using nasopharyngeal specimens (median Cp, 21.8 [range, 14.2 to 33.5]) was 100%, while that of both assays using saliva specimens (median Cp, 24.7 [range, 14.6 to 36.6]) was 97.1% (Table 3). All 122 specimens with moderate to high viral loads (Cp < 30) tested positive for SARS-CoV-2, while 92.3% (24/26) specimens with low viral loads (Cp ≥ 30) tested positive for SARS-CoV-2 (see Table S1 in the supplemental material).

**TABLE 3:** Diagnostic performance of the in-house-developed nsp8 and N gene RT-LAMP-CRISPR assays compared to the LightMix E gene RT-PCR assay for SARS-CoV-2 detection

Molecular assay	Molecular assay result	LightMix E gene RT-PCR		Sensitivity (%)	Kappa value (95% CI)^*a*^	*P* value by McNemar’s test
		Positive	Negative			
nsp8 RT-LAMP-CRISPR						
Nasopharyngeal specimens^*b*^	Positive	78	0	100	1	1
	Negative	0	71			
Saliva specimens^*c*^	Positive	68	0	97.1	0.976 (0.942–1)	0.5
	Negative	2^*e*^	100			
All specimens^*d*^	Positive	146	0	98.6	0.987 (0.97–1)	0.5
	Negative	2^*e*^	171			
N RT-LAMP-CRISPR						
Nasopharyngeal specimens^*b*^	Positive	78	0	100	1	1
	Negative	0	71			
Saliva specimens^*c*^	Positive	68	0	97.1	0.976 (0.942–1)	0.5
	Negative	2^*e*^	100			
All specimens^*d*^	Positive	146	0	98.6	0.987 (0.97–1)	0.5
	Negative	2^*e*^	171			

a CI, confidence interval.

b Median Cp, 21.8 (range, 14.2 to 33.5).

c Median Cp, 24.7 (range, 14.6 to 36.6).

d Median Cp, 23.1 (range, 14.2 to 36.6).

e The Cp values were 36.2 and 36.6.

**FIG 1:**
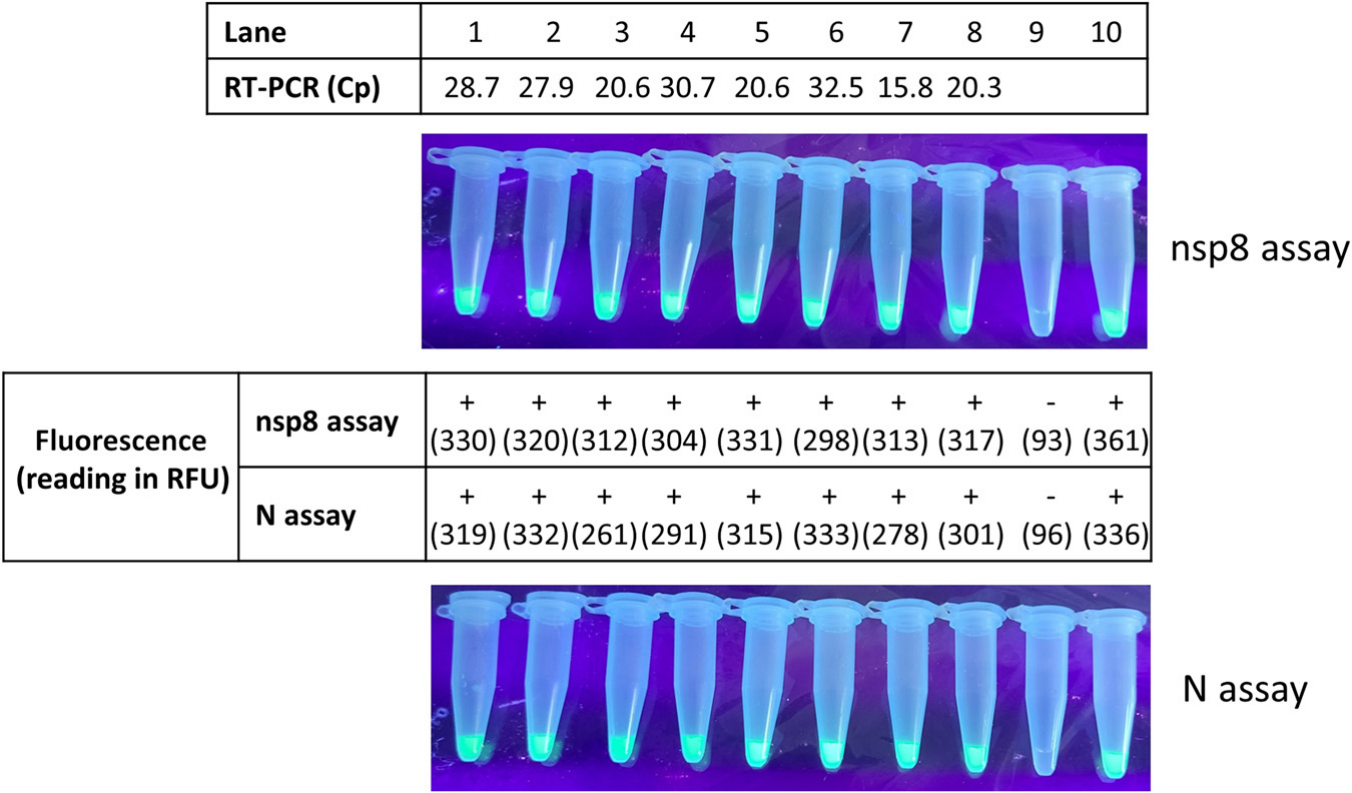
Detection of Omicron variants by the nsp8 and N gene RT-LAMP-CRISPR assays. Lanes 1 to 8, extracts of clinical specimens containing Omicron variants confirmed by nanopore sequencing; lane 9, water (negative control); lane 10, culture isolate extract (positive control). RFU, raw fluorescence unit.

The lineages of SARS-CoV-2 in the 146 positive specimens were identified by nanopore sequencing. The isolates belonged to variants of concern (Alpha, Beta, Gamma, Delta, and Omicron) and variants being monitored (Eta, Kappa, Mu, and Theta) (see Table S2 in the supplemental material). Our nsp8 and N gene RT-LAMP-CRISPR assays were able to detect the recently emerged Omicron variants as they showed green fluorescence for the eight clinical specimens (median Cp, 24.3 [range, 15.8 to 32.5]) (Fig. 1). For the two specimens that tested negative by the nsp8 and N RT-LAMP-CRISPR assays but positive by LightMix E gene RT-PCR, the lineage of SARS-CoV-2 could not be identified, which was probably due to the low viral load that was revealed by the high Cp values (36.2 and 36.6) for these two specimens (Table 3). Among the 3 proficiency testing (PT) samples from the College of American Pathologists (CAP) and the 8 PT samples from Quality Control for Molecular Diagnostics (QCMD), both the nsp8 and N gene RT-LAMP-CRISPR assays provided 100% correct results.

**FIG 2:**
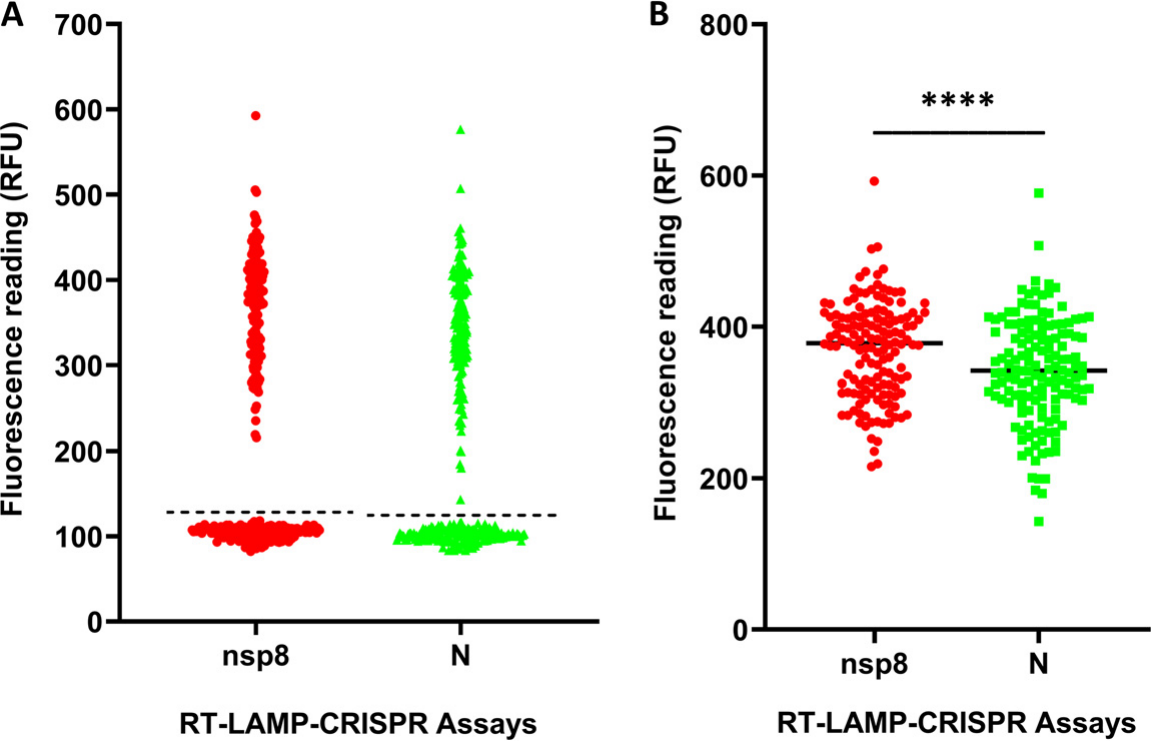
Fluorescence readings generated by the in-house-developed nsp8 and N gene RT-LAMP-CRISPR assays. Shown are 319 specimens tested by RT-LAMP-CRISPR assays, with the dashed threshold lines for nsp8 and N assays (A), and 146 specimens that tested positive by RT-LAMP-CRISPR assays (B). ****, *P* < 0.0001. RFU, raw fluorescence unit.

For the qualitative detection of SARS-CoV-2 RNA in this study, we visualized green fluorescence under UV excitation and measured the fluorescence intensity by a fluorometer for each reaction. The cutoff of fluorescence measured by the fluorometer was determined by calculating the mean plus 3 standard deviations of the fluorescence readings of the specimens that tested negative by LightMix E gene RT-PCR. The cutoff values for the nsp8 and N gene RT-LAMP-CRISPR assays were 126.3 RFU and 123.2 RFU, respectively (Fig. 2). Among the 319 clinical specimens, 148 specimens tested positive by LightMix E gene RT-PCR, 146 of which showed fluorescence readings above the cutoff values, while the fluorescence readings of the 171 specimens that tested negative by E gene RT-PCR were below the cutoff values. For the two specimens that tested positive by E gene RT-PCR but negative by our RT-LAMP-CRISPR assays, their fluorescence readings were between 96.9 RFU and 107 RFU. Among the 146 clinical specimens that tested positive by our RT-LAMP-CRISPR assays, the median fluorescence reading generated from the nsp8 assay (378.3 RFU [range, 215.6 to 592.6]) was significantly higher than that from the N assay (342.0 RFU [range, 143.0 to 576.6]) (*P* < 0.0001) (Fig. 2). Green fluorescence could also be easily visualized with the naked eye for both nasopharyngeal and saliva specimens under UV excitation (see Fig. S3 in the supplemental material).

**FIG 3:**
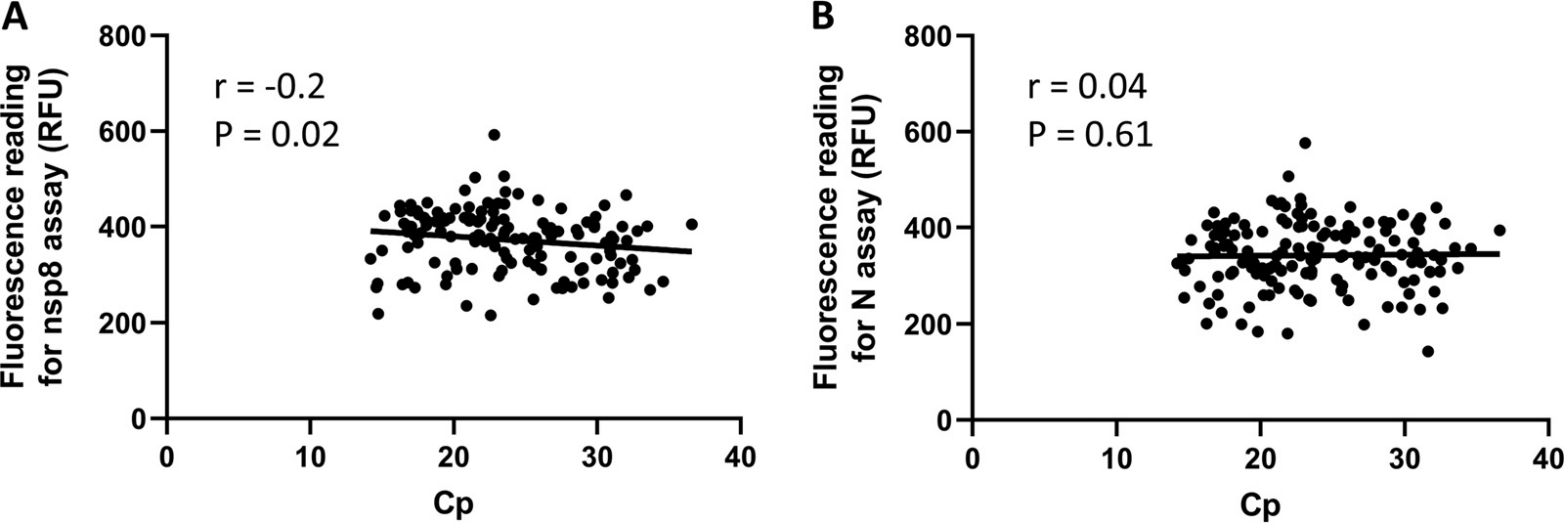
Correlation of the fluorescence readings generated by the nsp8 (A) and N (B) gene RT-LAMP-CRISPR assays and the Cp values from LightMix E gene RT-PCR. RFU, raw fluorescence unit.

To determine if there is any correlation between the fluorescence readings of RT-LAMP-CRISPR assays and the Cp values of LightMix E gene RT-PCR, scatterplots were drawn (Fig. 3). A weak correlation was noted between the fluorescence readings of the nsp8 assay and the Cp values of E gene RT-PCR (*r* = −0.2; *P* = 0.02), while there was no correlation between the fluorescence readings of the N gene RT-LAMP-CRISPR assay and the Cp values of LightMix E gene RT-PCR (*r* = 0.04; *P* = 0.61).

### Diagnostic performance of RT-LAMP-CRISPR assays without nucleic acid extraction.

As viral nucleic acid extraction is an extra step that takes time and requires specialized skill, we attempted to test our RT-LAMP-CRISPR assays using nasopharyngeal swab (NPS) and saliva specimens directly without nucleic acid extraction. First, we determined the optimal sample treatment and sample volume required for the reaction. We found that the RT-LAMP-CRISPR assay was negative when the volume of the NPS specimen was 5 μL, while 1 μL of the NPS specimen without heat pretreatment and 1 μL of saliva with heat pretreatment (98°C for 5 min) were the optimal conditions for our RT-LAMP-CRISPR assays (see Fig. S4 in the supplemental material).

Next, we evaluated a total of 28 specimens, including 14 NPS and 14 saliva specimens. The sensitivities of the nsp8 or N gene RT-LAMP-CRISPR assay were 27.3% (3/11) for NPSs and 54.5% (6/11) for saliva specimens (see Fig. S4 in the supplemental material). All specimens that tested negative by RT-PCR also tested negative by the nsp8 or N gene RT-LAMP-CRISPR assay. The specimens that tested positive by the nsp8 or N gene RT-LAMP-CRISPR assay had real-time RT-PCR Cp values of ≤22.3 for NPS and ≤25.8 for saliva specimens.

## DISCUSSION

In this study, we developed low-cost one-tube RT-LAMP-CRISPR assays for COVID-19 diagnosis. The one-tube format is made possible by depositing the CRISPR reagent mixture into the lid of the tube, as described previously (24), and therefore does not require opening of the tube during the assay process, which significantly reduces the risk of contamination due to amplicon carryover. Several studies have described similar approaches with similar sensitivities and specificities, but the reagent costs of our RT-LAMP-CRISPR assays, including enzymes, primers, deoxynucleoside triphosphate (dNTP), MgSO_4_, Cas12a protein, gRNA, and the single-stranded DNA (ssDNA) reporter, were less than $2 per reaction, which is lower than the costs of the RT-LAMP-CRISPR assays reported by others (22, 24, 25) since smaller amounts of RT-LAMP reagents were used in our study. RT-LAMP reagents are quite inexpensive, unlike those for other isothermal amplification formats like recombinase polymerase amplification (RPA) assays for which a commercial kit is not available and that require the addition of reverse transcriptase from another source, increasing the costs (26, 27).

We used primers targeting the nsp8 and N genes for two separate RT-LAMP-CRISPR assays. Due to the ongoing emergence of new variants with mutations scattered across the genome, it is always advisable to design primers targeting different genes. The SARS-CoV-2 N gene is a common target for COVID-19 NAATs, but N gene target failure has been reported (28, 29). In our previous study, the nsp8 gene was abundantly expressed in clinical samples (20), which makes this an attractive alternative conserved target gene for COVID-19 diagnosis. Our assays could detect all evaluated variants of concern (including the recently emerged Omicron) and variants under investigation. In the present study, we showed that the median fluorescence reading from the nsp8 assay was significantly higher than that from the N gene assay, demonstrating that the nsp8 assay allows better visualization, especially for samples with low viral loads when observing fluorescence by the naked eye. However, since there was a weak correlation between the nsp8 RT-LAMP-CRISPR fluorescence readings and the RT-PCR Cp values, the RT-LAMP-CRISPR assay is not suitable for quantitative analysis.

The sensitivity for saliva specimens was 97.1%, which was lower than that for NPS specimens. This is likely related to the low viral loads in these saliva specimens. The Cp values for the saliva specimens that tested positive by RT-PCR but negative by our RT-LAMP-CRISPR assays were 36.2 and 36.6. For the nasopharyngeal specimens, all positive specimens had a Cp value of <34, and therefore, we are not able to determine the sensitivity for NPS specimens with a Cp value of ≥34.

Our RT-LAMP-CRISPR assays were evaluated using sample extracts, but nucleic acid extraction takes time and requires specialized skill to perform. Therefore, we tried to use direct clinical specimens to test our assays. During assay optimization, we found that no fluorescence was detected for the reaction using 5 μL of a direct specimen (the same volume as that for the viral nucleic acid that we used for testing); this may be due to the presence of inhibitors in clinical specimens when larger sample volumes were used. A stronger signal could be detected when smaller volumes (1 or 2 μL) of direct specimens were used. Several previous studies suggest different lysis methods, such as by heat or lysis buffer/proteinase K treatment (30–32). We did not use lysis buffer/proteinase K in this evaluation because of the extra cost and inconvenience compared to heat pretreatment, so we only compared the samples with and without heat pretreatment. Improvements in SARS-CoV-2 detection using samples by heating them at 95°C for 10 min or 98°C for 5 min before direct-to-test addition have been reported (31, 32); we chose the latter one because of the shorter time. Interestingly, heat pretreatment improved SARS-CoV-2 detection for saliva specimens but not for NPS specimens. This finding was consistent with those of another study that demonstrated a significant increase in the detection sensitivity when using saliva samples with prolonged heat pretreatment (33). Finally, 1 μL of NPSs without heat pretreatment and 1 μL of saliva with heat pretreatment at 98°C for 5 min were the optimal conditions for RT-LAMP-CRISPR assays. We then further evaluated 14 NPS and 14 saliva specimens with various concentrations of SARS-CoV-2 or that were negative for SARS-CoV-2 by our RT-LAMP-CRISPR assays using the optimized conditions. Although a lower detection sensitivity was noted for the assays using direct specimens than for the assays using purified sample extracts, it is interesting to note that our assays using direct saliva specimens with heat pretreatment showed a higher detection sensitivity than that of assays using direct NPS specimens. Nevertheless, the use of direct specimens can help reduce the time and cost compared to viral nucleic acid extraction, and it is particularly useful when there is a shortage of extraction reagents.

Although real-time RT-PCR is the most common method for COVID-19 diagnosis due to its high sensitivity and specificity, RT-LAMP-CRISPR assays carry considerable advantages over RT-PCR assays. Our RT-LAMP-CRISPR assays performed with sensitivity and specificity comparable to those of the real-time RT-PCR assay. These assays do not require real-time PCR systems, which are bulky and expensive. Indeed, they can be performed on simple heating blocks (21, 24). The equipment cost of real-time RT-PCR is >45-fold higher than that of RT-LAMP (34). Furthermore, the running time of RT-LAMP-CRISPR assays is less than an hour, which is shorter than those of RT-PCR assays (14, 35–37). The fluorescence readout enables the visual interpretation of results, although this can be enhanced by using a fluorometer. The advantage of using a fluorometer over the naked eye is that measurement by a fluorometer is an objective readout of the fluorescence, which eliminates the subjectiveness of using the naked eye. We did not measure the fluorescence intensity with a cell phone. However, with advances in technology, it should be possible to measure fluorescence using a cell phone in the future.

The RT-LAMP-CRISPR assay (fluorometric approach) has several advantages over the colorimetric RT-LAMP assay. First, the colorimetric assay has a higher chance of giving false-positive results than the fluorometric assay. The colorimetric assay relies on the change in the pH, and therefore, a specimen with a lower pH can lead to false-positive results in the colorimetric assay (38, 39). Second, elution buffers for viral RNA extraction could significantly affect colorimetric readings, such as false-negative results; this may be due to the pH effect of or the chemicals in these buffers (40), while the elution buffer that we used for extraction did not have an adverse effect on our RT-LAMP-CRISPR assays. Third, colorimetric interpretation was time sensitive for samples, including the negative control, which could turn positive when the incubation time was more than 40 min for the RT-LAMP reaction (41, 42). Fourth, the colorimetric assay does not involve the use of gRNA, and thus, there is a higher chance of the detection of nonspecific products (41). To overcome this problem, the addition of Cas protein and gRNA specific for a viral gene target can enhance the specificity of the fluorometric assay. Fifth, a fluorometric assay with higher detection sensitivity than a colorimetric assay has been reported (43).

A key component of our newly developed assay is the use of CRISPR-Cas12a chemistry to detect products of the RT-LAMP reaction. Cas12a has distinct advantages over other Cas-associated proteins used for diagnostic applications, such as Cas3, Cas9, Cas12b, or Cas13a (44–48). Cas12b was used for CRISPR reactions in several studies, but it requires >100-nucleotide (nt)-long gRNA for the reaction (44, 45). Since longer gRNA has a risk of partial overlap between the gRNA and one of the primers for RT-LAMP, this may lead to false-positive results due to sporadic collateral activity (24, 44). Besides, the cost of longer synthetic RNA is higher. In our study, we used Cas12a for CRISPR, and it requires ~40-nt-long gRNA for the reaction. Hence, the chance of false-positive results can be reduced. Moreover, the Cas12a protein that we used in our study is commercially available, so the concentration and quality of the protein are guaranteed. Furthermore, unlike Cas13a, Cas12a does not require the additional transcription of the DNA into RNA.

In summary, our RT-LAMP-CRISPR assays for detecting the SARS-CoV-2 nsp8 and N genes are sensitive, specific, affordable, fast, and easy to perform. As the COVID-19 pandemic continues, we believe that such assays have considerable value in resource-limited settings to improve the COVID-19 diagnostic capacity.

## MATERIALS AND METHODS

### Viruses, clinical specimens, and proficiency testing of samples for evaluation.

For analytical sensitivity evaluation, 2-fold serial dilutions of total nucleic acid (TNA) extracted from SARS-CoV-2 Q control 01 with a target concentration of 10,000 copies/mL (Qnostics, UK) were used. Triplicates were performed for each dilution in two independent experiments.

For analytical specificity evaluation, TNAs extracted from the culture isolates of human coronaviruses (SARS-CoV-1, Middle East respiratory syndrome coronavirus [MERS-CoV], human coronavirus OC43 [HCoV-OC43], HCoV-229E, and HCoV-NL63), influenza A viruses [A(H1N1)pdm09 and H3N2], influenza B virus, influenza C virus, human adenovirus, rhinovirus, respiratory syncytial virus, human metapneumovirus, and human parainfluenza virus types 1 to 4 were used. For HCoV-HKU1, TNA was extracted from a patient’s specimen (49–51).

For the evaluation of diagnostic performance, archived respiratory specimens that were previously tested by a real-time RT-PCR assay using the LightMix SarbecoV E gene kit (TIB MolBiol, Germany) from 2020 to 2021 were included in this study. A total of 319 clinical specimens (149 nasopharyngeal and 170 posterior oropharyngeal saliva specimens) from 319 hospitalized patients (male/female ratio, 149/170; median age, 35 years [range, 9 months to 103 years]) with suspected COVID-19 were selected for SARS-CoV-2 RNA detection. The SARS-CoV-2-positive specimens were subjected to lineage identification by nanopore sequencing (52). In addition to clinical specimens, 11 proficiency testing samples from the CAP and QCMD with different concentrations of SARS-CoV-2 RNA or that were negative for SARS-CoV-2 RNA were also evaluated.

This study was approved by the Institutional Review Board of the University of Hong Kong/Hospital Authority Hong Kong West Cluster (HKU/HA HKW IRB) (UW 20-224). Since archived specimens were used, written informed consent was waived.

### *In silico* analysis.

Multiple-sequence alignment was performed by ClustalX 2.1 using our primer and guide RNA (gRNA) sequences and the nsp8 gene sequences of SARS-CoV-2 variants (ancestral strain, Alpha, Beta, Gamma, Delta, Omicron, Lambda, and Mu) from different geographical regions. The ancestral strain sequence (GenBank accession number NC_045512.2) and the sequences of other variants were obtained from the NCBI GenBank database or the Global Initiative on Sharing All Influenza Data (GISAID) EpiCoV database.

### Reverse transcription–loop-mediated isothermal amplification.

TNA extraction was performed using the MagNA Pure 96 extraction system (Roche, Switzerland) according to the manufacturer’s instructions, as we described previously (7). Briefly, 200 μL of each sample was mixed with MagNA Pure 96 external lysis buffer (Roche). After extraction, TNA was recovered in 50 μL of elution buffer and then kept at −80°C until use.

Before the preparation of a master mix for the reverse transcription–loop-mediated isothermal amplification (RT-LAMP) assay, 10× LAMP primer mix was prepared (see Table S3 in the supplemental material). The RT-LAMP reagent mixture (10 μL) contained 0.4 μL of nuclease-free water, 1 μL of 10× isothermal amplification buffer (NEB, USA), 0.6 μL of 100 mM MgSO_4_ (NEB), 1.4 μL of 10 mM dNTP solution mix, 1 μL of 10× LAMP primer mix, 0.4 μL of *Bst* 2.0 WarmStart DNA polymerase (8,000 U/mL) (NEB), 0.2 μL of WarmStart RTx reverse transcriptase (15,000 U/mL) (NEB), and 5 μL of TNA as the template. RT-LAMP reactions were performed at 60°C for 40 min for the COVID-19 nsp8 assay, at 62°C for 30 min for the COVID-19 N assay, and at 62°C for 40 min for the human RNase P assay.

### CRISPR Cas12a-based fluorescence detection.

A Cas12a *trans*-cleavage assay was performed using methods similar to those described in a previous study by Pang et al. (24). Briefly, the reagent mixture (10 μL) contained 1.7 μL of nuclease-free water, 2 μL of 10× NEBuffer r2.1, 1 μL of 10 μM gRNA, 0.8 μL of 10 μM EnGen Lba Cas12a (Cpf1) (NEB), 0.5 μL of 100 μM ssDNA reporter, and 4 μL of 100 mM MgSO_4_. The gRNAs specifically targeting the RT-LAMP amplicons of the nsp8 and N genes of SARS-CoV-2 and the human RNase P gene and the ssDNA reporter (Table 4) were synthesized by IDT.

**TABLE 4:** The guide RNAs and ssDNA reporter used for CRISPR in this study^*a*^

gRNA (reference[s]) or ssDNA reporter	Sequence (5′–3′)
SARS-CoV-2_nsp8_gRNA	UAAUUUCUACUAAGUGUAGAUCUUAUGCAUCAGCAUUGUGGGA
SARS-CoV-2_N_gRNA (21, 24)	UAAUUUCUACUAAGUGUAGAUCCCCCAGCGCUUCAGCGUUC
RNaseP-POP7_gRNA (21)	UAAUUUCUACUAAGUGUAGAUAAUUACUUGGGUGUGACCCU
ssDNA reporter	FAM-TTATTATT-IABkFQ

a ssDNA, single-stranded DNA; FAM, 6-carboxyfluorescein. IABkFQ, Iowa Black Fluorescent Quencher.

### One-tube RT-LAMP-CRISPR assays for SARS-CoV-2 detection.

Ten microliters of the RT-LAMP reagent mixture was added to the bottom of a 0.5-mL PCR tube (Sarstedt, Germany), and 10 μL of the Cas12a reagent mixture was added inside the cap of the tube (see Fig. S5 in the supplemental material). Five microliters of TNA was added to the bottom of the tube and mixed with the RT-LAMP reagent by pipetting up and down. The tube was gently capped and put in a thermocycler (Eppendorf, Germany) without closing the lid, and the bottom of the tube was kept at the optimal temperature for 30 to 40 min for the RT-LAMP reaction. When the RT-LAMP reaction was completed, the tube was flicked with the wrist, and the Cas12a reagent mixture was mixed with the RT-LAMP amplicon. The tube was then incubated in the thermocycler at a constant temperature of 37°C for 10 min. Samples that showed green fluorescence under the excitation of a UV lamp were regarded as positive, and photos were taken using a smartphone. The fluorescence intensity was also measured using a Qubit 4 fluorometer (Invitrogen, USA). Samples that showed a fluorescence intensity above the cutoff value were regarded as positive.

### Real-time RT-PCR assay for SARS-CoV-2 detection.

The LightMix SarbecoV E gene kit (TIB MolBiol, Germany) with LightCycler multiplex RNA virus master mix (Roche) was used according to the manufacturer’s instructions. Each 20-μL reaction mixture consisted of 5.4 μL of nuclease-free water, 0.5 μL of reagent mix, 4 μL of Roche master mix, 0.1 μL of RT enzyme, and 10 μL of TNA as the template. RT-PCR was performed using a LightCycler 480 II real-time PCR system (Roche). The thermal cycling conditions were 55°C for 3 min and 95°C for 30 s, followed by 45 cycles of 95°C for 3 s and 60°C for 12 s.

### Statistical analysis.

The kappa statistic was used to determine the agreement between the in-house-developed assay and the reference method. McNemar’s test was used to compare the performance of the in-house-developed assays with that of the reference method. The fluorescence readings of the two RT-LAMP-CRISPR assays were compared using a Wilcoxon signed-rank test. Spearman’s correlation was used to assess the relationship between the fluorescence readings from RT-LAMP-CRISPR assays and the Cp values from LightMix E gene RT-PCR. A *P* value of <0.05 was considered statistically significant. Statistical analysis was performed using GraphPad Prism 9 or IBM SPSS Statistics 26.
