# Supplementary material for: Development and Validation of a Novel COVID-19 nsp8 One-Tube RT-LAMP-CRISPR Assay for SARS-CoV-2 Diagnosis
Source: Microbiol Spectr. 2022 Nov 29;10(6):e01962-22. doi: 10.1128/spectrum.01962-22 (PMC9769742; doi:10.1128/spectrum.01962-22)
Supplement: Supplemental file 1 — Supplemental material. Download spectrum.01962-22-s0001.pdf, PDF file, 1.5 MB [file spectrum.01962-22-s0001.pdf]

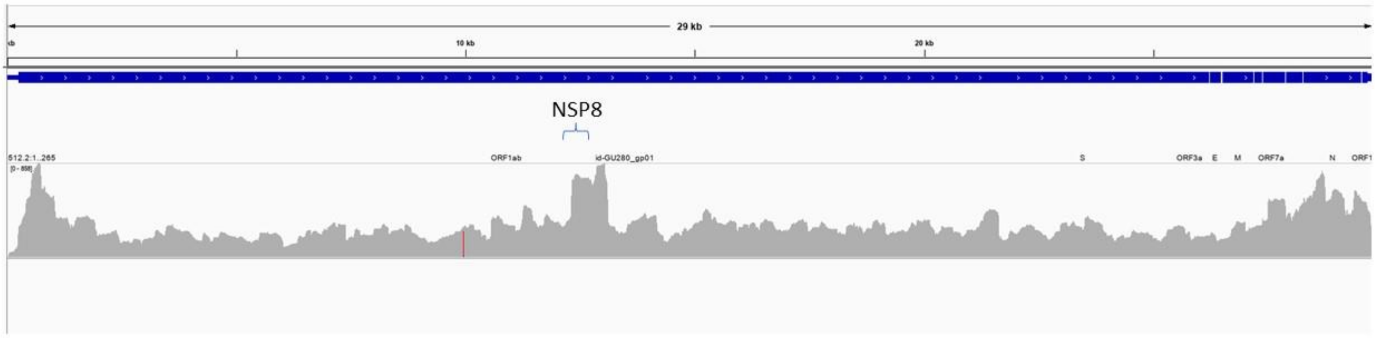

FIG S1. Whole genome coverage map of a SARS-CoV-2 sample. The map was exported from Integrative Genomics Viewer (IGV). The X-axis represents the nucleotide position with reference to the reference sequence, NC_045512.2, while the Y-axis shows the coverage with respect to each position. The horizontal bracket indicates the position of NSP8 on the SARS-CoV-2 genome.

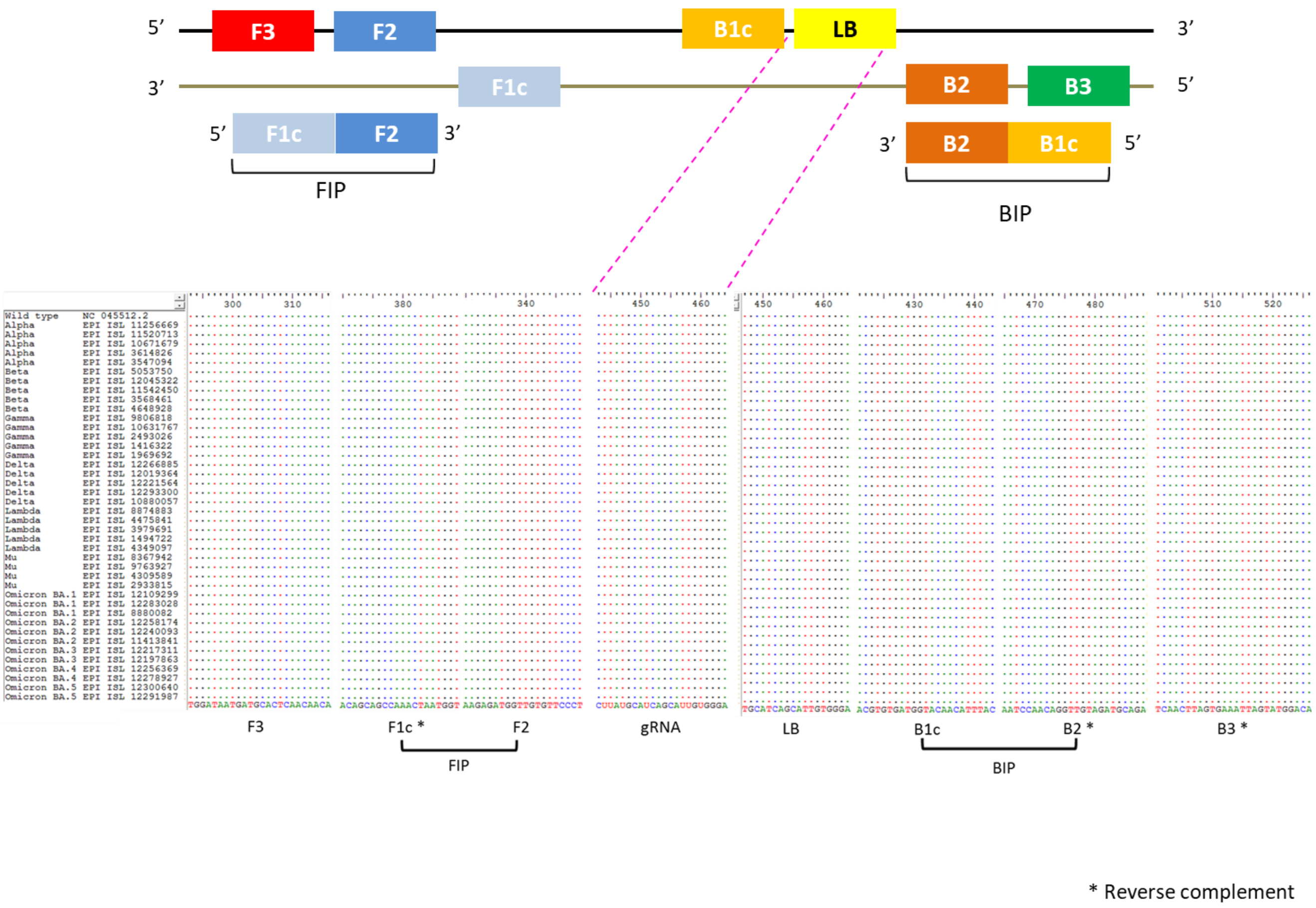

FIG S2. Multiple sequence alignment using our primer and gRNA sequences and the nsp8 gene sequences of SARS-CoV-2 variants (ancestral strain, Alpha, Beta, Gamma, Delta, Omicron, Lambda and Mu) from different geographical regions. Pink dotted lines indicate the gRNA region.

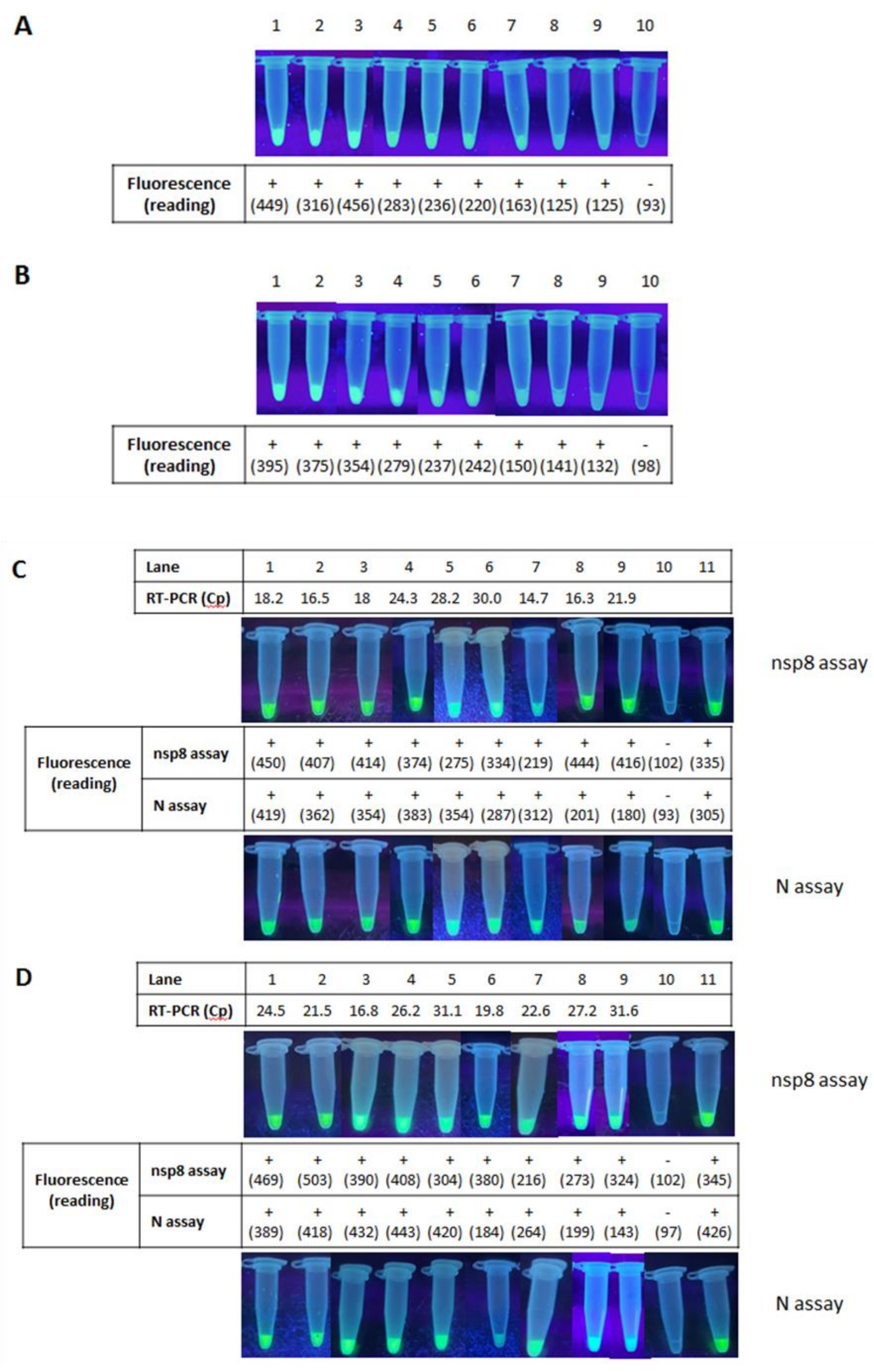

FIG S3. Detection of human RNase P gene by RT-LAMP-CRISPR assay. Nasopharyngeal specimens (A); saliva specimens (B). Lane 1-9 clinical specimen extracts; lane 10, water (negative control). Visualization of green fluorescence generated from RT-LAMP-CRISPR assays for both nasopharyngeal (C) and saliva (D) specimens with different viral loads of SARS-CoV-2. Lane 1-9, clinical specimen extracts; lane 10, negative control; lane 11, culture isolate extract (positive control). Fluorescence readings were in raw fluorescence units (RFU).

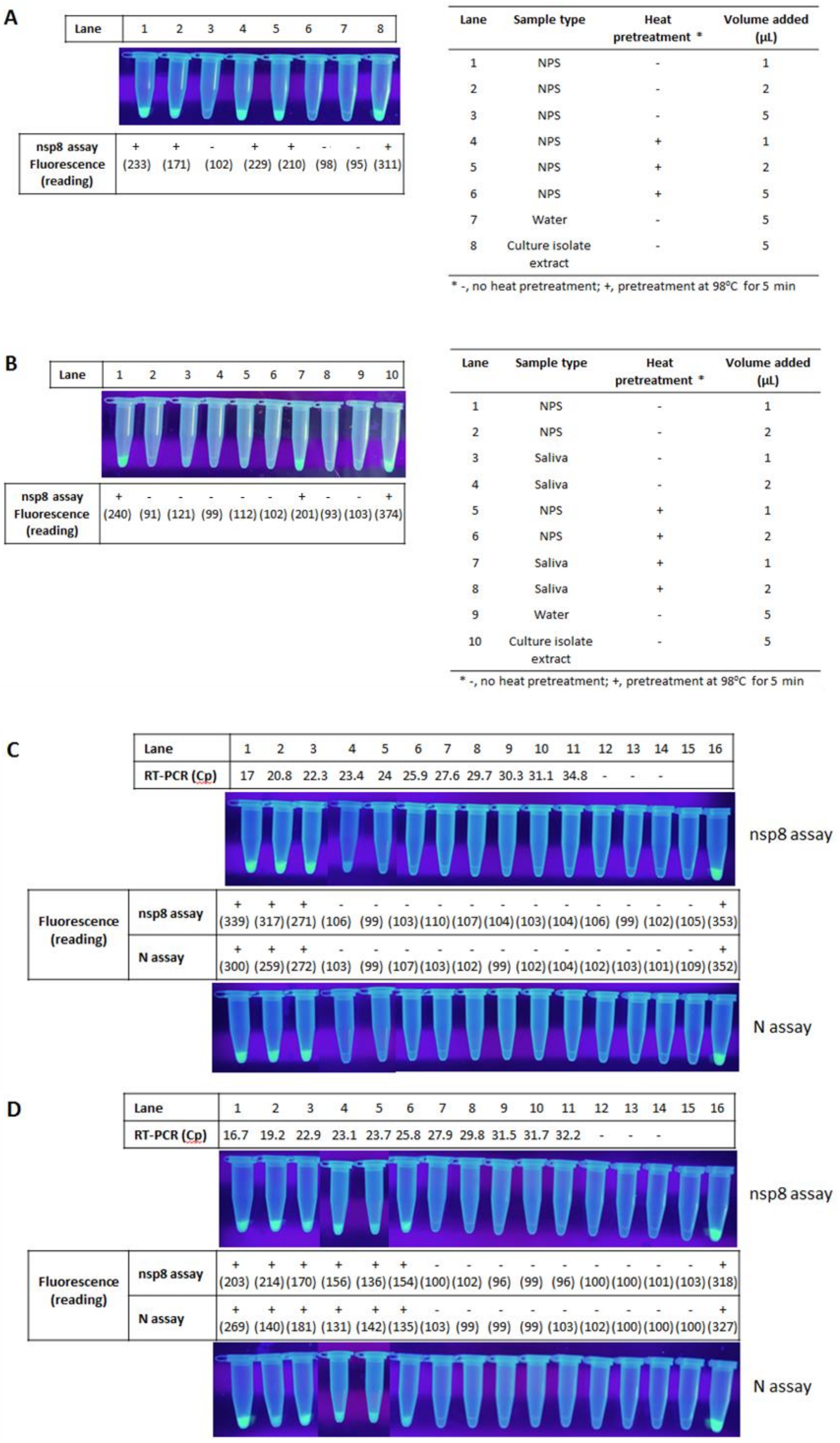

FIG S4. RT-LAMP-CRISPR assay optimizations using a direct NPS specimen with high viral load (Cp: 17.0) by adding different volumes of the sample with or without heat pretreatment (A), and using direct NPS (Cp: 22.3) and saliva (Cp: 22.9) specimens with moderate viral loads by adding different volumes of the specimens with or without heat pretreatment (B). Detection of SARS-CoV-2 by the nsp8 and N gene RT-LAMP-CRISPR assays using direct NPS (C) and saliva (D) specimens. Lane 1-14, clinical specimens; lane 15, negative control; lane 16, positive control. Fluorescence readings were in raw fluorescence units (RFU).

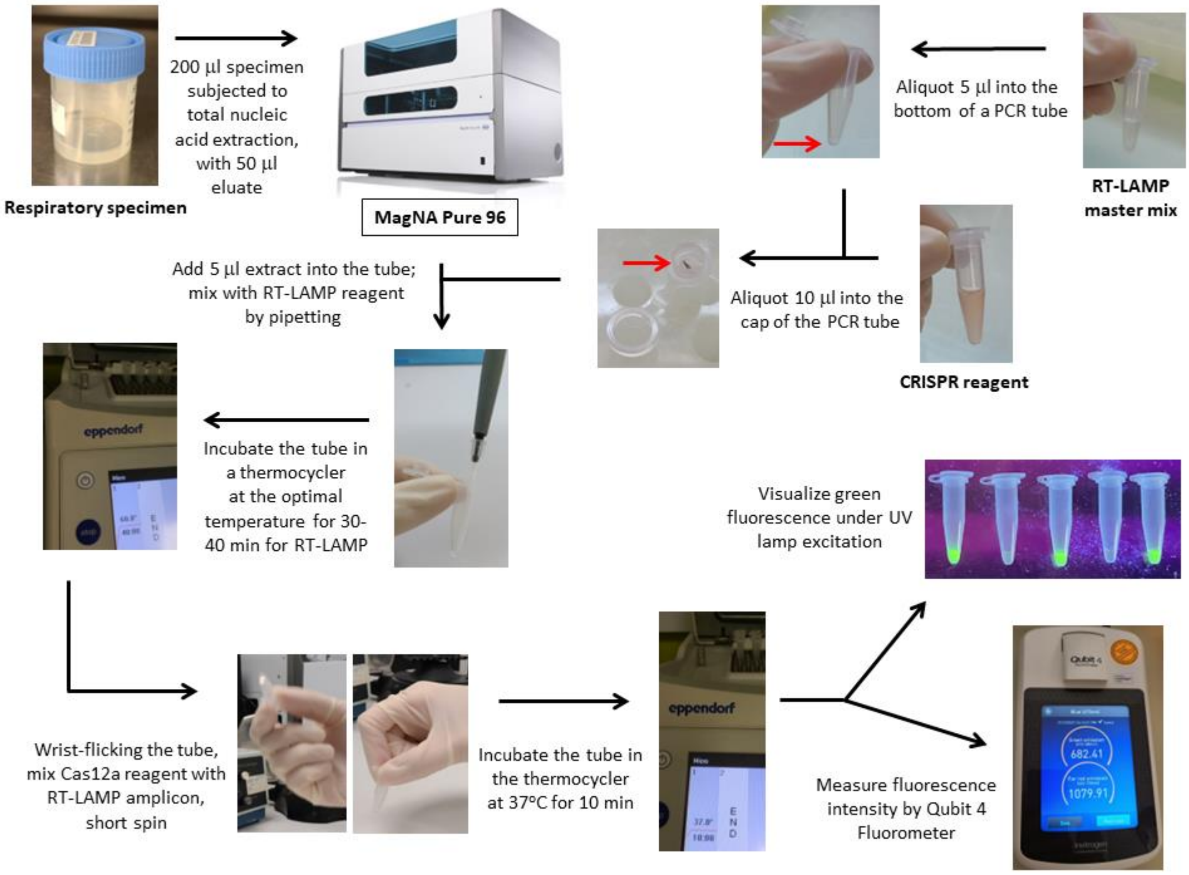

FIG S5. A workflow of RT-LAMP-CRISPR assays in this study.

TABLE S1 Number of SARS-CoV-2-positive specimens for each Cp range by E-gene RT-PCR

**Specimen type**	**Number of specimens**				
	Cp < 25	Cp ≥ 25 – 29.99	Cp ≥ 30 – 34.99	Cp ≥ 35	**Total**
Nasopharyngeal specimens	53	14	11	0	78
Saliva specimens	36	19	12	3*	70
All specimens	89	33	23	3*	148

*Two saliva specimens tested negative by our RT-LAMP-CRISPR assays

TABLE S2 Variants or lineages of SARS-CoV-2 identified in clinical specimens

VOC / VBM*	Number
Alpha	10
Beta	8
Gamma	1
Delta	40
Omicron	8
Eta	3
Kappa	13
Mu	1
Theta	2

*Variants of Concern / Variants being Monitored (from WHO)

Pango Lineage	Number
A	1
A.21	1
AY.3	1
B	1
B.1	5
B.1.1	3
B.1.1.529	8
B.1.1.63	6
B.1.1.7	10
B.1.177	2
B.1.210	1
B.1.351	7
B.1.351.3	1
B.1.36	5
B.1.36.27	19
B.1.459	3
B.1.466.2	5
B.1.470	3
B.1.480	1
B.1.525	3
B.1.562	1
B.1.617.1	13
B.1.617.2	39
B.1.621	1
B.43	1
B.6	2
P.1	1
P.3	2
Untypeable	2

TABLE S3 Preparation of 10X LAMP Primer Mix (for 100 reactions)

10X LAMP Primer Mix for nsp8:

	Volume (μL)
Nuclease-free water	60
SARS-CoV-2_nsp8_FIP (100 μM)	16
SARS-CoV-2_nsp8_BIP (100 μM)	16
SARS-CoV-2_nsp8_F3 (100 μM)	2
SARS-CoV-2_nsp8_B3 (100 μM)	2
SARS-CoV-2_nsp8_LB (100 μM)	4

10X LAMP Primer Mix for N:

	Volume (μL)
Nuclease-free water	48
SARS-CoV-2_N_FIP (100 μM)	16
SARS-CoV-2_N_BIP (100 μM)	16
SARS-CoV-2_N_F3 (100 μM)	2
SARS-CoV-2_N_B3 (100 μM)	2
SARS-CoV-2_N_LF (100 μM)	8
SARS-CoV-2_N_LB (100 μM)	8

10X LAMP Primer Mix for RNase P:

	Volume (μL)
Nuclease-free water	56
RNaseP-POP7_FIP (100 μM)	16
RNaseP-POP7_BIP (100 μM)	16
RNaseP-POP7_F3 (100 μM)	2
RNaseP-POP7_B3 (100 μM)	2
RNaseP-POP7_LF (100 μM)	4
RNaseP-POP7_LB (100 μM)	4

SUPPLEMENTARY DATA GISAID sequences acknowledgement table

We gratefully acknowledge the following Authors from the Originating laboratories responsible for obtaining the specimens, as well as the Submitting laboratories where the genome data were generated and shared via GISAID, on which this research is based.

All Submitters of data may be contacted directly via www.gisaid.org

Authors are sorted alphabetically.

Acknowledgement EPI_SET Identifier: EPI_SET_20220511an

Accession ID	Originating Laboratory	Submitting Laboratory	Authors
EPI_ISL_12278927	ASP Reggio Calabria Polo Sanitario Nord - Dr.ssa Fiorillo	SOC Microbiologia e Virologia - AO Pugliese-Ciaccio	Pasquale Minchella
EPI_ISL_1494722, EPI_ISL_3568461	Area of Virology, Serology and Virology Division (SAViD), New South Wales Health Pathology Randwick	Virology Research Laboratory; Area of Virology, Serology and Virology Division (SAViD), New South Wales Health Pathology Randwick	Au, J.; Bull, R.; Deveson, I.; Foster, C.; Rawlinson, W.; Ruiz Silva, M.; Van Hal, S.
EPI_ISL_12217311	Broad Institute Clinical Research Sequencing Platform	Infectious Disease Program, Broad Institute of Harvard and MIT	Adams, G.; B.L.; B.W.; Bauer, M.; Birren; Blumenstiel, B.; Brown, C.; Carter, A.; Chaluvadi, S.; D.J.; DeFelice, M.; DeRuff, K.; Dodge, S.; Gabriel, S.; Gallagher, G.; Gladden-Young, A.; Granger, B.; J.E.; K.J.; Lagerborg, K.; Larkin, K.; Lee, M.; Lemieux; Lennon, N.; Loreth, C.; Madoff, L.; McGovern, S.; Meldrim, J.; Normandin, E.; P.C.; Park; Pearlman, L.; Reilly, S.; Rudy, M.; Sabeti; Siddle; Smole, S.; Tomkins-Tinch, C.; Vicente, G.; and MacInnis
EPI_ISL_12291987	Department of Bacteria, Parasites and Fungi, Statens Serum Institut, Copenhagen, Denmark	Statens Serum Institut Bioinformatics and Microbial Genomics	Danish Covid-19 Genome Consortium
EPI_ISL_11413841	Department of Microbiology, The University of Hong Kong	Department of Microbiology, The University of Hong Kong	Kelvin K.W. To; Kwok-Yung Yuen
EPI_ISL_9806818	EXCITE Lab	Andersen lab at Scripps Research	Abigail Schnapper; Angela Scioscia; Cheryl Anderson; Chip Schooley; Greg Humphrey; Helena Tubb; Natasha Martin; Sawyer Farmer; Smruthi Karthikeyan; Tommy Valles + SEARCH
EPI_ISL_1969692	Guangdong Provincial Center for Disease Control and Prevention	National Institute for Viral Disease Control and Prevention, China CDC	Baisheng; Min Kang; Xiang Zhao; Xiaoling Deng; Yang Song; Yao Hu; Zhencui Li
EPI_ISL_10671679	Helen Joseph Hospital	National Institute for Communicable Diseases of the National Health Laboratory Service	Amoako DG; Bhiman JN; Everatt J; Ismail A; Kekana D; Mahlangu B; Mnguni A; Mohale T; Ntuli N; Scheepers C; Wolter N
EPI_ISL_12300640	Helix	Centers for Disease Control and Prevention Division of Viral Diseases, Pathogen Discovery	Benjamin Rambo-Martin; Christopher Gulvick; Clinton Paden; Dakota Howard; Dhwani Batra; Duncan MacCannell; Erisa Sula; Helix CA; Jason Caravas; Kristine Lacek; Matthew Schmerer; Peter Cook; Scott Sammons; Shatavia Morrison; Tymeckia Kendall; Victoria Caban Figueroa; Yvette Unoarumhi
EPI_ISL_4648928	Hong Kong Department of Health	School of Public Health, The University of Hong Kong	Daniel K.W. Chu; Dominic N.C. Tsang; Haogao Gu; Leo L.M. Poon; Malik Peiris; Tong Zhang
EPI_ISL_12109299	Houston Methodist Hospital	Houston Methodist Hospital	Akanksha Batajoo; James J. Davis; James M. Musser; Jessica Cambric; Jimmy Gollihar; Jordan Pachuca; Kristina Reppond; Madison N. Shyer; Matthew Ojeda Saavedra; Nicole Kanellopoulos; Paul A. Christensen; Randall J. Olsen; Rashi M. Thakur; Regan Mangham; Richard Snehal; Robert Olson; Ryan Gadd; S. Wesley Long; Sindy Pena; Sinjini Gupta; Yuvanesh Vedaraju
EPI_ISL_9763927	INHRR	Laboratorio de Virologia Molecular	Carmen L Loureiro; CoViMol Group; Domingo J Garzaro; Flor H Pujol; Héctor R Rangel; José Luis Zambrano; Lieska Rodríguez; Mariana Hidalgo; Pierina D´Angelo; Rossana C Jaspe; Víctor Alarcón; Yoneira Sulbaran; Zoila Moros
EPI_ISL_2493026	Instituto Nacional de Investigação em Saúde	CERI, Centre for Epidemic Response and Innvoation, Stellenbosch University and KRISP, KZN Research Innovation and Sequencing Platform, UKZN.	Afonso P; David K; Emmanuel SJ; Freitas RH; Giandhari J; Inglês L; Lutucuta S; Miranda J; Morais J; Mufinda M; Naidoo Y; Neto Z; Paulo A Carralero RR Paixão JP; Pereira A; Pillay S; Tegally H; Wilkinson E; de Oliveira T
EPI_ISL_12197863	Labor Dr. Krause & Kollegen MVZ GmbH Kiel	Robert Koch Institute	
EPI_ISL_4309589	Laboratoire de Recherche et d'Analyses Medicales de la Gendarmerie Royale	Laboratoire de Recherche et d'Analyses Medicales de la Gendarmerie Royale	Amal SOUIRI; Hajar LEMRISS; Mohamed LABIOUI; Nabil LEMZAOUI; Sanaâ LEMRISS; Saâd EL KABBAJ
EPI_ISL_8874883	Laboratorio de Referencia Nacional de Virus Respiratorios. Centro Nacional de Salud Publica. Instituto Nacional de Salud Peru.	Laboratorio de Referencia Nacional de Virus Respiratorios. Centro Nacional de Salud Publica. Instituto Nacional de Salud Peru.	Alicia Nuñez Llanos; Carlos Padilla Rojas; Edwart Rogger Rivera Serrano; Henri Bailon Calderon; Iris Silva Molina; Joseph Huayra Niquen; Kelly Vanessa Izarra Rojas; Lely Solari Zerpa; Luis Barcena Flores; Marco Galarza Perez; Nancy Rojas Serrano; Nieves Sevilla Castañeda; Omar Caceres Rey; Orson Mestanza Millones; Princesa Medrano Alhuay; Priscila Lope Pari; Sara Gordillo Vilchez; Steve Acedo Lazo; Veronica Hurtado Vela; Victor Jimenez Vasquez; Wendy Lizarraga Olivares
EPI_ISL_8367942	Laboratory Corporation of America	Centers for Disease Control and Prevention Division of Viral Diseases, Pathogen Discovery	Amanda Douglas; Amanda Suchanek; Andrea Throop; Ayla Burns; Benjamin Rambo-Martin; Bobbi Croy; Brian Krueger; Brian Norvell; Christopher Gulvick; Christos Petropoulos; Clinton Paden; Craig Lukasik; Dakota Howard; Debbie Boles; Dhwani Batra; Duncan MacCannell; Eyad Almasri; Goran Stevovic; Howard Engler; Hrushikesh Deshmukh; Jake Humphrey; Jana Schroth; Jason Caravas; Joe Voshell; John Pruitt; Jonathan Meltzer; Jonathan Williams; Kimberly Wagner; Kristine Lacek; Lax Iyer; Lisa Pfefferle; Lyndon Tilson; Manoj Jain; Marcia Eisenberg; Mary Cristobal; Mary Williamson; Matthew Robinson; Matthew Schmerer; Michael Levandoski; Mike Sapeta; Mindy Nye; Minoo Agarwal; Mohan Kolli; Nuthawin Charoensri; Oren Cohen; Peter Cook; Prashant Gupta; Qian Zeng; Rama Ghatti; Scott Parker; Scott Ryan; Scott Sammons; Shatavia Morrison; Stanley Letovsky; Steven Ragan; Suresh Selvaraju; Susan Countryman; Susan Hicks; Suzanne Dale; Thomas Urban; Tim Kuphal; Tricia Zwiefelhofer; Tymeckia Kendall; Victoria Caban Figueroa; Vincent Drouillon; Yvette Unoarumhi
EPI_ISL_12240093	Laboratory of Clinical Microbiology, Virology and Bioemergencies, ASST Fatebenefratelli Sacco - Sacco Hospital	Laboratory of Clinical Microbiology, Virology and Bioemergencies, ASST Fatebenefratelli Sacco - Sacco Hospital	Valeria Micheli
EPI_ISL_12293300	Lifebrain Covid Labor GmbH	Lifebrain Covid Labor GmbH	Abhishek Mitra; Alexandra Wagner; Filip Sima; Florian Scharhauser; Hannes Hagen; Kristina Bavrka Kolenc; Lucia Castello; So Jung Han
EPI_ISL_12283028	Lighthouse Lab in Glasgow	Wellcome Sanger Institute for the COVID-19 Genomics UK (COG-UK) Consortium	Anna Dominiczak and Alex Alderton; Carol Clugston; Cordelia Langford; David Gray; David K. Jackson; Dominic Kwiatkowski; Ewan Harrison; Harper VanSteenhouse; Ian Johnston; Jeffrey Barrett; John Sillitoe on behalf of the Wellcome Sanger Institute COVID-19 Surveillance Team; Roberto Amato; Sonia Goncalves; Yumi Kasai
EPI_ISL_12019364	Limbach - MVZ Labor Dr. Volkmann & Kollegen	Robert Koch Institute	
EPI_ISL_12045322	Max von Pettenkofer Institute, Virology, National Reference Center for Retroviruses, LMU Munich	Laboratory for Functional Genome Analysis; Dept. Genomics; Gene Center of the LMU Munich	Alexander Graf; Helmut Blum; Max Muenchhoff; Oliver Keppler; Stefan Krebs
EPI_ISL_4475841	Microbiology Department, University Hospital Donostia	Microbiology Department, University Hospital Donostia	Cilla G.; Gomez M; Marimon JM; Martin-Peñaranda T; Montes M; Piñeiro L; Sorarrain A
EPI_ISL_12221564	Mowbray Maternity Hospital wc MMH	NHLS/UCT	Arash Iranzadeh; Carolyn Williamson; Diana Hardie; Gert Marais; Innocent Mudau; Luicer Olubayo; Marvin Hsiao; Nokuzola Mbhele; Rageema Joseph; Stephen Korsman
EPI_ISL_3979691	Outre Mer	National Reference Center for Viruses of Respiratory Infections, Institut Pasteur, Paris	Angela Brisebarre; Camille Capel; Christophe Malabat; Corinne Maufrais; Etienne Simon-Lorière; Frédéric Lemoine; Julien Fumey; Louise Lefrançois; Marion Barbet; Maud Vanpeene; Méline Bizard; Patrice Combe; Slim El-Khiari; Sylvie Behillil; Sylvie Van der Werf; Vincent Enouf
EPI_ISL_12256369, EPI_ISL_12258174	Pandemic Response Lab - NYC	Pandemic Response Lab, R&D	Alex Carpio; Cybill del Castillo; Haiping Hao; Isabel Fernandez Escapa; Jon Laurent; Melissa Hopkins; Michael Hammerling; Simran Chhabria; Simran Gupta; Sol Rey; Steven Chase; Tiara Rivera; William Ward
EPI_ISL_1416322	PathWest Laboratory Medicine WA	PathWest Laboratory Medicine WA Microbial Surveillance Unit	PathWest Laboratory Medicine WA Microbial Surveillance Unit
EPI_ISL_8880082	Princess Margaret Hospital	Hong Kong Department of Health	Alan K.L. Tsang; Edman T.K. Lam; Ken H.L. Ng; Patricia K. L. Leung; Peter C.W. Yip; Rickjason C.W. Chan
EPI_ISL_12266885	Public Health Ontario Laboratory	Public Health Ontario Laboratory	Aimin Li; Alex Marchand-Austin; Andre Villegas; Anna Puzinovici; Ashleigh Sullivan; Brandon Ye; Candice Schreiber; Carla Duncan; Christina Rampertab; Christine Seah; Claudia Chu; Dean Maxwell; Dhiraj Gaglani; Doonia Bajovic; Esther Nagai; Fatemeh Shaeri; Fatima Merza; Grace Jeong; Hadia Hussain; Himeshi Samarsinghe; Jacob Afelskie; Jason Iraheta; Jesse Wang; John Palmer; Karthikeyan Sivaraman; Kirby Cronin; Lisa Kim; Lisa McTaggart; Maria Mariscal; Mark Horsman; Narisha Shakuralli; Nataliya Potapova; Natasha Sing; Nobish Varghese; Philip Banh; Rachelle DiTullio; Rebecca Azzaro; Rima Palencia; Samir N Patel; Sarah Teatero; Semra Tibebu; Sophie Yu; Surendra Kumar; Sushma Kavikondala; Vincent Su Bin Cha; Zarah Rajaei
EPI_ISL_3614826	Queensland Health Forensic and Scientific Services	Queensland Health Forensic and Scientific Services	Chenwei Wang on behalf of Q-PHIRE Genomics
EPI_ISL_10631767, EPI_ISL_11520713	Respiratory Virus Unit, Microbiology Services Colindale, Public Health England	COVID-19 Genomics UK (COG-UK) Consortium	PHE Covid Sequencing Team
EPI_ISL_2933815, EPI_ISL_4349097	SARS-CoV-2 testing team,	Pathogen Genomics Center, National	Hazuka Y Furihata; Hiromizu Takahashi; Kentaro Itokawa; Makoto Kuroda; Masanori Hashino; Masumichi Saito; Naomi Nojiri; Nozomu Hanaoka; Rina Tanaka; Tsuguto Fujimoto; Tsuyoshi Sekizuka
